# Supplementary material for: Role of donor nerves in supercharge end-to-side nerve transfer: A rat model study of varying injury severity
Source: PLoS One. 2026 Apr 1;21(4):e0346252. doi: 10.1371/journal.pone.0346252 (PMC13042668; doi:10.1371/journal.pone.0346252)
Supplement: Attachment — Submitted filename: review Comments.pdf [file pone.0346252.s002.pdf]

Comments to the authors

Thanks for your effort and contribution in such literature gap

Abstract :

Line no 19- your work is much similar to SETS performed for high ulnar injury and to lesser amount severe entrapment with axonal damage

Introduction :

Paragraph starting from line 48 till 55
You are concentrating on cubital tunnel decompression rather traumatic high ulnar nerve injuries although your study is mimicking traumatic injury and to lesser extent cubital tunnel decompression
It would be much better if u reversed the paragraph

Material and methods :

Line 89
Why you chose to transect the peroneal nerve in the control group ?

Line 146 Immunohistochemistery

How did you obtained nerve tissue from the three sites at 8 weeks without affecting the later on regeneration process

As you stated that the immunohistochemistery photos and count are in the axial plane .

Line 208 :
It should be stated in the methods first that recovery rate will be tested after donor nerve transection rather than to be first stated in the results section

Line 226: muscle weight ratio

How did u obtain the muscle weight in 8 weeks
In the methods u stated that the ratio was done at the sacrifice
So how to be done at 8 weeks then repeated at 16 weeks ?

And how do you explain there is difference in the weight ratio in the control group although the tibial nerve is intact

Table 2 data are confusing .

How do you explain that peroneal nerve NF count is lower in the severe SET - and + groups at 16 weeks time in comparison to control and mild although the difference is in the tibial nerve

Also how the NF count in the severe sets group at distal tibial nerve dropped from 8 weeks (4957) to 3962 at 16 weeks

Discussion

Line 309 in mild cubital tunnel syndrome by definition there is no axonal changes only velocity and latency are affected so in mild entrapment cases there is no justification for any distal SETS .

So your results and recommendation in mild cases could be referred in traumatic cases with more distal sites or small nerve gaps or direct primary repair not in mild entrapment .

Line 320-322
I think it should be reversed
As your study is focusing and mimiking nerve gaps reconstruction and to lesser amount severe cubital tunnel

Line 330 331
What is the transitional relevance ??
