# Supplementary material for: Role of donor nerves in supercharge end-to-side nerve transfer: A rat model study of varying injury severity
Source: PLoS One. 2026 Apr 1;21(4):e0346252. doi: 10.1371/journal.pone.0346252 (PMC13042668; doi:10.1371/journal.pone.0346252)
Supplement: Attachment — Submitted filename: Response to Reviewers.docx [file pone.0346252.s003.docx]

**Response to Reviewers**

**Title of the Manuscript:** Role of donor nerves in supercharge end-to-side nerve transfer: a rat model study of varying injury severity

**Manuscript Number:** PONE-D-25-55769

We sincerely thank the Academic Editor and the reviewers for their careful evaluation of our manuscript and for their constructive and insightful comments. We have revised the manuscript accordingly, and all changes are highlighted in the revised version with track changes. Our detailed responses are provided below.

Review Comments to the Author:

**Reviewer #1:**

Using a rat model with different degrees of nerve injury, the authors showed that donor nerves in supercharge end-to-side (SETS) transfer function differently according to the severity of recipient nerve damage.

Their functional and histological findings indicate that SETS can accelerate early recovery in mild injuries and support long-term regeneration in more severe cases, possibly through a temporary “babysitting” effect on distal targets.

The study is well designed, the data are internally consistent, and the conclusions logically follow from the results.

Response: We thank the reviewer for the positive and encouraging comments.

**Reviewer #2:**

Dear Author,

I had the opportunity to review this study. The constructs and study design are appropriately described and are methodologically sound. The study is well designed, with thoughtfully selected groups and proper implementation of the procedures. The statistical analysis is appropriate and correctly applied.

The Results are presented in a clear and readable manner.

The Discussion section effectively interprets the findings and relates them to the existing literature.

Overall, the manuscript addresses an interesting and relevant research topic and represents a very engaging and valuable contribution to the field.

The manuscript is written in a clear, concise, and focused style and is easy to read. The Introduction adequately introduces the topic, clearly presents the research problem, and outlines the study objectives.

Minor comments:

Figure captions should be placed in a separate section after the References rather than within the main text. This would improve the clarity of the manuscript and help distinguish the main text from figure descriptions.

Response: We appreciate this helpful suggestion. We reviewed the PLOS ONE formatting guidelines and confirmed that figure captions may be placed within the main text. Accordingly, we retained the captions in their current position in accordance with the journal requirements. In addition, we have revised the captions to improve clarity and readability.

The figures are of low quality and difficult to read; improvement is required.

Response: We have revised all figures to improve clarity by increasing resolution and refining font presentation, ensuring readability at high magnification in accordance with PLOS ONE requirements.

Once again, I congratulate you on a well-prepared manuscript.

**Reviewer #3:**

Comments to the authors

Thanks for your effort and contribution in such literature gap

Abstract :

Line no 19- your work is much similar to SETS performed for high ulnar injury and to lesser amount severe entrapment with axonal damage

Response: Thank you for this important observation. We agree that our experimental model shares similarities with conditions such as proximal nerve injury and, to a lesser extent, compressive neuropathy with axonal involvement. While the clinical motivation of this study was related to conditions such as cubital tunnel syndrome, we acknowledge that the current experimental model more closely reflects incomplete nerve injury. We have revised the Abstract to better reflect this interpretation and to clarify the potential clinical relevance of our findings.

Introduction :

Paragraph starting from line 48 till 55

You are concentrating on cubital tunnel decompression rather traumatic high ulnar nerve injuries although your study is mimicking traumatic injury and to lesser extent cubital tunnel decompression

It would be much better if u reversed the paragraph

Response: Thank you for this helpful suggestion. We have revised the paragraph to first describe the application of SETS in proximal nerve injuries, followed by its clinical use in cubital tunnel syndrome, to improve logical flow and consistency with the experimental model.

Material and methods :

Line 89

Why you chose to transect the peroneal nerve in the control group ?

Response: The peroneal nerve was transected to eliminate potential donor nerve influence and to isolate the effect of the recipient tibial nerve. This clarification has been added to the Methods section.

Line 146 Immunohistochemistery

How did you obtained nerve tissue from the three sites at 8 weeks without affecting the later on regeneration process

As you stated that the immunohistochemistery photos and count are in the axial plane .

Response: We have clarified in the Experimental design section that tissue samples at 8 and 16 weeks were obtained from separate animals and harvested only at the time of sacrifice; therefore, the regeneration process was not affected. All histological analyses were performed on transverse (axial) sections.

Line 208 :

It should be stated in the methods first that recovery rate will be tested after donor nerve transection rather than to be first stated in the results section

Response: A description of recovery rate analysis after donor nerve transection has been added to the Methods section, and the Results section has been revised accordingly.

Line 226: muscle weight ratio

How did u obtain the muscle weight in 8 weeks

In the methods u stated that the ratio was done at the sacrifice

So how to be done at 8 weeks then repeated at 16 weeks ?

Response: We have clarified in the Experimental design section that muscle weight measurements at 8 and 16 weeks were performed in separate animals at the time of sacrifice.

And how do you explain there is difference in the weight ratio in the control group although the tibial nerve is intact

Response: We agree with the reviewer’s observation. The slight difference in muscle weight ratio in the control group may be attributable to minor surgical manipulation, such as nerve exposure and handling during the procedure, even though the tibial nerve itself was not transected. The tibial nerve was exposed in the control group in the same manner as in the other groups. This likely reflects procedural variability rather than a true physiological effect.

Table 2 data are confusing .

Response: We appreciate the reviewer’s comment. To improve clarity, Table 2 has been revised by separating the NF-positive axon counts and S100β-positive Schwann cell counts into distinct sections, and by presenting each data point on a single line to improve readability. These changes enhance the overall clarity of the table without altering the original data.

How do you explain that peroneal nerve NF count is lower in the severe SET - and + groups at 16 weeks time in comparison to control and mild although the difference is in the tibial nerve

Also how the NF count in the severe sets group at distal tibial nerve dropped from 8 weeks (4957) to 3962 at 16 weeks

Response: We appreciate the reviewer’s careful evaluation of Table 2. In the severe model, the number of NF-positive axons in the distal tibial nerve decreased from 8 to 16 weeks. This may reflect progressive distal degeneration associated with prolonged denervation, which is known to impair Schwann cell support and limit axonal regeneration (Sulaiman and Gordon, 2000).

Regarding the lower NF counts in the peroneal nerve in the severe groups at 16 weeks, we consider that this may reflect a change in axonal distribution. Regenerating axons do not necessarily distribute uniformly and may show preferential reinnervation toward specific pathways (Brushart, 1998). Because this mechanism was not directly tested in the present study, we have presented this point as a plausible explanation rather than a definitive conclusion. These points have been clarified in the Discussion, with the above references added.

Discussion

Line 309 in mild cubital tunnel syndrome by definition there is no axonal changes only velocity and latency are affected so in mild entrapment cases there is no justification for any distal SETS .

So your results and recommendation in mild cases could be referred in traumatic cases with more distal sites or small nerve gaps or direct primary repair not in mild entrapment .

Line 320-322

I think it should be reversed

As your study is focusing and mimiking nerve gaps reconstruction and to lesser amount severe cubital tunnel

Response: We thank the reviewer for this important and insightful comment. We agree that clinically defined mild cubital tunnel syndrome typically does not involve significant axonal degeneration and therefore may not justify distal SETS.

In response, we have revised this section of the Discussion to clarify that the mild model used in this study does not directly correspond to mild compressive neuropathy, and that our findings in the mild model may be more applicable to conditions involving partial axonal injury, such as distal nerve injury or small nerve gaps.

We have also reorganized the paragraph to emphasize nerve reconstruction and regeneration as the primary context of this study, rather than cubital tunnel syndrome. Severe cubital tunnel syndrome is now presented as one example of impaired regenerative conditions, which better aligns with the experimental design.

In addition, we have moderated our statements regarding the use of SETS in mild conditions, indicating that while early functional support may be observed, routine use is not justified when spontaneous regeneration is sufficient.

Line 330 331

What is the transitional relevance ??

Response: We have revised this expression to improve clarity by replacing “translational relevance” with a more specific description of the limitation. The revised text clarifies that the short nerve length in rodents may facilitate local axonal sprouting, which may differ from clinical conditions involving longer regeneration distances.
